# Development and Piloting of a Mental Health Prevention and Referral Program for Veterans and Their Families in Ukraine

**DOI:** 10.9745/GHSP-D-22-00488

**Published:** 2023-06-21

**Authors:** Amanda J. Nguyen, Tara Russell, Stephanie Skavenski, Sergiy Bogdanov, Kira Lomakina, Iryna Ivaniuk, Luke R. Aldridge, Paul Bolton, Laura Murray, Judy Bass

**Affiliations:** a School of Education and Human Development, University of Virginia, Charlottesville, VA, USA.; b Independent contractor, County Cork, Ireland.; c Johns Hopkins Bloomberg School of Public Health, Baltimore, MD, USA.; d National University of Kyiv-Mohyla Academy, Kyiv, Ukraine.

## Abstract

**Background:**

While growing evidence exists for the effectiveness of mental health interventions in global mental health, the evidence base for psychosocial supports is lacking despite the need for a broader range of supports that span the prevention–treatment continuum and can be integrated into other service systems. Following rigorous evaluation of the Common Elements Treatment Approach (CETA) in Ukraine, this article describes the development and feasibility testing of CETA Psychosocial Support (CPSS), a brief psychosocial prevention and referral program for Ukrainian veterans and their families.

**CPSS Development:**

CPSS development used evidence-based CETA intervention components and was informed by a stakeholder needs analysis incorporating feedback from veterans and their families, literature review, and expert consultations. The program includes psychoeducation, cognitive coping skill development, and a self-assessment tool that identifies participants for potential referral. After initial development of the program, the intervention underwent: (1) initial implementation by skilled providers focused on iterative refinement; (2) additional field-testing of the refined intervention by newly trained providers in real-world conditions; and (3) a formal pilot evaluation with collection of pre-post mental health assessments and implementation ratings using locally validated instruments.

**Results:**

Fifteen CPSS providers delivered 14 group sessions to 109 participants (55 veterans, 39 family members, and 15 providers from veterans’ service organizations). After incorporating changes related to content, process, and group dynamics, data from the pilot evaluation suggest the refined CPSS program is an acceptable and potentially effective brief psychosocial prevention and promotion program that can be implemented by trained veteran providers. Forty percent of participants required safety or referral follow-ups.

**Conclusion:**

The iterative, inclusive development process resulted in an appropriate program with content and implementation strategies tailored to Ukrainian veterans and their families. Brief psychosocial programs can fit within a larger multitiered mental health and psychosocial continuum of care that supports further referral.

## Abstract (precis)

The CETA Psychosocial Support program is a single-session intervention that can be delivered by lay providers to support mental health prevention and referral for veterans and their families in Ukraine.

## Teaser

**Key Findings:**

- The Common Elements Treatment Approach Psychosocial Support (CPSS) program was developed with iterative feedback from Ukrainian veterans and their families to normalize veterans’ stress and distress, develop skills in cognitive coping, and provide referral to more intensive mental health services for those at high risk for suicide and/or with a higher burden of symptoms.
- The development process resulted in a mental health and psychosocial support prevention and promotion program that was designed to be implemented in nonclinical environments to reduce barriers to mental health services and to be integrated into different health and social service systems to support referral to more intensive treatment, as needed.
- The CPSS program is an acceptable and potentially effective brief psychosocial prevention program that can be implemented by trained veteran providers in Ukraine.

**Key Implications:**

- Psychosocial support (PSS) programs should be developed based on input from multiple stakeholders, including the target population and providers.
- PSS programs need to be part of a broader continuum of the mental health and psychosocial care system, including referral pathways for mental health services for high-risk cases and those who need more mental health services.

## BACKGROUND

The past 2 decades have seen monumental growth in recognition and understanding of the global burden of mental illness.^1^ Calls and challenges to develop innovative approaches to address the mental health treatment gap have focused on building the evidence base for mental health interventions, addressing human resource constraints in mental health service delivery, and increasing policy and funding prioritization for mental health services and service systems.^2^ As a result of these efforts, substantial evidence now exists for the fit and effectiveness of numerous mental health treatments, often delivered by nonspecialist providers through task-sharing strategies.^3^

In light of the growth of mental health treatments, a shift is needed to develop promotive and preventive interventions^4^ that can address population-wide mental health needs using a public health approach that calls for a continuum of care.^5^ In lower-resource and humanitarian settings, this continuum is often described under the umbrella term “mental health and psychosocial support” (MHPSS).^6^ Within the MHPSS continuum, preventive and promotive interventions are typically less focused or resource intensive, allowing for delivery to a larger proportion of the population to reduce the burden on more resource-intensive treatments. However, findings from recent systematic reviews highlight the lack of systematic and rigorous development and evaluation of most preventive and promotion programs,^7^ which limits the ability to create effective multitiered systems of care for addressing mental health problems and promoting mental well-being.^2^

To continue advancing progress in addressing mental health treatment gaps, global mental health practitioners and researchers must develop and evaluate psychosocial support (PSS) prevention and promotion programs that can be delivered in different settings, such as clinics, community centers, schools, and shelters, and that include identification and referral of individuals in need of more targeted mental health treatments.^4^ Knowledge of and uptake of mental health services continues to be a challenge, particularly in unstable contexts and with stigma-sensitive subgroups.^8^ Integrating PSS programs into a broader MHPSS care continuum can address some of these barriers by improving case finding and increasing access to mental health interventions for those who need more targeted treatment while reducing stigma, avoiding fragmentation, and strengthening health system capacity.^4^ An approach that strengthens mental health promotive factors could also reduce the future need for higher-level mental health services.

## COMMON ELEMENTS TREATMENT APPROACH

We previously encountered the need for integrated approaches in Ukraine during a trial of a psychotherapeutic treatment, the Common Elements Treatment Approach (CETA), with Ukrainian veterans and internally displaced populations.^8^ Developed specifically to address the treatment gap in low-resource settings, CETA was designed to (1) address multiple and often co-occurring mental health problems, (2) address problems across the spectrum of need from prevention to treatment, and (3) work across the life span (youth to elderly).^9^ CETA uses a modular treatment approach, where both content and length of CETA elements and sessions can vary depending on the type and severity of presenting problems of the CETA participant. The CETA intervention has been found effective in reducing symptoms of depression, anxiety, posttraumatic stress, interpersonal violence, and alcohol use with multiple populations in different countries and contexts.^8^^,^^10^^–^^12^ While trial results similarly indicated strong efficacy for the intervention in Ukraine,^8^ we also observed an overall low level of help-seeking behaviors and difficulty engaging clients in treatment, particularly among war veterans and their families. This highlighted the need to develop a program that could focus on prevention and promotion behaviors and supports, as well as stronger pathways to care for those in need of CETA treatment.

The continuing implementation and scale-up of CETA in Ukraine after completion of the trial provided the opportunity to build a broader CETA care system in alignment with the MHPSS care continuum. Our goal was to develop a relatively light-touch psychosocial program using CETA elements that could be integrated into existing community-level health and social service systems, serving as both a preventive and promotive intervention and an accessible entry point for referral into further mental health care for those in need. The ability to implement programming within community-based systems was particularly important because Ukraine was similar to countries where the mental health system remains highly centralized, and a strong reliance on psychiatric inpatient services contributes to substantial mental health stigma and low help-seeking. As such, we coordinated with both the Ukraine Ministry of Health and Ministry of Veterans Affairs (MOVA) to ensure the resulting program would have the potential for service integration.

Our goal was to develop a light-touch psychosocial program using CETA elements that could be integrated into existing community-level health and social service systems.

In this article, we describe the development and piloting of this CETA Psychosocial Support (CPSS) prevention and promotion program that includes referral to full CETA treatment for use with Ukrainian veterans and their families. The program, supported by the U.S. Agency for International Development, was designed for implementation in nonclinical environments to introduce MHPSS programming and content in a more familiar and acceptable setting. To support mental health promotion, the program provides participants with skills to increase positive coping to stress while also identifying individuals that would benefit from more intensive mental health treatment or who need immediate safety supports. Those who are identified as needing more services would be referred to existing mental health services, including counseling with CETA-trained counselors. Using a systems approach framework, we intended that the CPSS program, once formally evaluated, would function as a first-tier intervention that could ultimately be integrated into many different health and social service systems to support referral linkages and decrease barriers to mental health services usage at higher tiers of the mental health care system.

## CPSS DEVELOPMENT PROCESS

Beginning in summer 2019, the CPSS intervention development followed an ADDIE (Analysis, Design, Development, Implementation, and Evaluation^13^) approach to program design and was guided by principles from the Johns Hopkins University’s DIME (Design, Implementation, Monitoring, and Evaluation^14^) model for applied mental health research, which places substantial emphasis on initial qualitative needs assessment to pragmatically inform local programming while also generating knowledge to inform future services. Specifically, we describe 5 phases of development: (1) stakeholder needs analysis; (2) design and initial development of the intervention prototype; (3) round 1 implementation and iterative refinement using rapid prototyping^15^ with skilled providers; (4) round 2 implementation to field-test the refined model with new providers; and (5) formal pilot evaluation with associated data collection. Stakeholder involvement, focus of inquiry, and related aspects of these phases are described in the Table.

**TABLE.** Key Aspects of CPSS Iterative Intervention Development in Ukraine

	**Stakeholder-Engaged Needs Analysis**	**Round 1 Implementation**	**Round 2 Implementation**	**Formal Pilot Study**
Timing	July–August, 2019	September 2019	October 2019	March 2020
Setting	Kyiv and Zaporhizya	Neutral setting	Community setting	Community setting
Stakeholders involved	80 veterans and family members; 58 stakeholders involved in providing supports to veterans and family members; additional representatives from MOVA, MOH, community organizations	62 participants (38 veterans, 9 family members, 15 service providers); implemented by 9 experienced CETA providers with observers from development team	23 new participants (9 veterans, 14 family members); 6 newly trained CPSS providers	24 new participants (8 veterans, 16 family members) Same 6 newly trained CPSS providers as round 2
Intervention	N/A	8 sessions averaging 109 minutes, with manipulation of group size and composition, examples, and individual vs. group exercises; follow-up phone call for safety indication	3 sessions averaging 111 minutes (with break); follow-up call within 1 week for all participants to review assessment results and skill practice	3 sessions averaging 133 minutes (with study procedures); 1-week follow-up calls, 1-month follow-up assessment and referrals
Focus of inquiry	Psychosocial support needs, barriers to help-seeking, potentially acceptable support strategies	Participant acceptability; necessary refinements to intervention	Provider acceptability; feasibility of training and delivery by community-based providers	Refine and practice study procedures; initial trends in outcomes and implementation domains
Information collected	Free lists and in-depth interviews	Focus group discussions; feedback from providers; developer observations	Focus group discussions; feedback from providers	Self-assessment data (pre-post); implementation survey (post)
Key findings	Potential of a brief PSS intervention embedded within trusted networks to strengthen coping skills and foster positive relationships to support referrals	Necessary changes to content (examples and activities); process (contextualization and length), and group dynamics (group activities); led to revised intervention and greater training emphasis on group facilitation	Increased participant acceptability; training and community-based implementation was feasible; continued development of fidelity tracking tools	Positive participant perspectives on implementation domains; positive trends in reduced symptoms and functional impairment; high 1-month attrition potentially due to COVID-19 outbreak

Abbreviations: CETA, Common Elements Treatment Approach; CPSS, CETA Psychosocial Support; MOH, Ministry of Health; MOVA, Ministry of Veterans Affairs; PSS, psychosocial support.

### Stakeholder Needs Analysis

After completion of the CETA trial,^8^ in July–August 2019, we engaged 80 veterans and their family members in Kyiv and Zaporizhya, as well as an additional 58 stakeholders (e.g., social workers, volunteers, lawyers, and heads of charitable foundations) considered to be knowledgeable of or engaged in providing supports to veterans, to explore what adults in Ukraine consider to be the main push and pull factors for veterans and their family members to engage with mental health support services. Stakeholders shared that social welfare programs, those providing psychosocial support and education, and programs provided by peers were among the most sought-after types and “accepted” programming for these groups. Data from the stakeholders indicated that veterans were motivated to seek services due to mental health problems related to conflict exposure, as well as family problems, substance use issues, feelings of social isolation, and problems related to aggression. In contrast, a lack of information, lack of trust, stigma, and shame were all identified as challenges to engaging veterans. Stakeholders described what they perceived as feasible strategies to overcome these barriers, including providing information about programs, involving peers who share similar lived experiences, and building relationships through a comfortable environment and respectful interactions. It was also mentioned as critical that participants quickly perceive some positive impact of the program. Finally, veterans reported primarily learning about and being directed to programs through word of mouth, social media groups, and information received through local veterans’ associations. These results strongly supported the potential of a brief PSS intervention embedded within trusted networks to strengthen coping skills and foster positive relationships to support referrals.

The needs analysis identified lack of information, lack of trust, stigma, and shame as challenges to engaging veterans.

During this planning phase, we also coordinated closely with both the Ministry of Health and MOVA and built collaborative relationships with a number of community-based veterans’ service organizations. We consulted with the MOVA on evidence-based approaches and stepped-care models, assessment tools, training, supervision models, low-intensity interventions, and safety protocols. We also met with representatives of both ministries to discuss components of a stepped-care model and how this might be implemented in the future, including integration into the MOVA’s planned “Veterans’ Hubs.” The MOVA and community organizations also provided venues for consultation and testing activities, as well as promoting these activities to the veteran community. The advice and guidance of these stakeholders were also instrumental in identifying the profile of a provider of the brief PSS intervention. Both the MOVA and community organizations were also invited to nominate individuals for the first trainings in the intervention who were currently working in positions in social services where they could integrate the intervention into their routine work with the veteran community.

#### Literature Review

Based on findings from the previous CETA trials in Ukraine and elsewhere regarding both CETA’s effectiveness and feasibility for delivery by lay providers, we planned to develop a brief intervention using CETA components. To optimize reach and ultimately embed the intervention into other veterans’ service activities, we developed a group-based delivery model. To inform intervention development, we reviewed the literature on PSS programming in conflict-affected settings^8^ and consulted with experts on best practices in brief intervention modalities,^16^ in addition to drawing on our team’s clinical expertise in implementation of modular CETA.^9^^,^^17^ In using the CETA content to develop a brief prevention and engagement program, we sought to address issues of content, format, and delivery.

#### Content

In terms of content, our team looked at maximizing effectiveness through practical skill-building, not just information delivery, and to support symptom reduction and mental health promotion. Our reviews and consultations also confirmed that the following were important: (1) assessment of symptoms (for potential referral), (2) safety assessment and planning if needed (i.e., for the risk of suicide or homicide), and (3) normalization of mental health symptoms (i.e., communicating that these symptoms are both common and treatable, and not an indication that someone is “crazy,” to promote engagement). We also recognized the need to avoid elements and activities that would require more clinical intervention; for example, although many veterans wanted to discuss traumatic experiences, this would not be an appropriate target for a brief, single-session intervention.

The team maximized effectiveness of the content through practical skill-building, not just information delivery.

#### Format

Regarding format, we carefully considered the length of the program and the size of the participant group. We had to carefully balance the brief format while retaining the content of the clinical elements. For example, whereas a single session would be more palatable, elements derived from cognitive behavioral therapy often incorporate after-session practice as a necessary component.^18^ We also weighed the pros and cons of a small versus larger group format. Smaller groups would allow for more direct interaction between providers and participants, while larger groups would enable greater reach. Ultimately, size consideration was primarily driven by a prioritization of being able to do appropriate safety risk identification, ensuring a size that would allow providers the ability to quickly screen and refer anyone reporting substantial risk of harm to self or others.

#### Delivery

Finally, delivery considerations included designing an intervention that could be facilitated by lay and peer providers—veterans themselves or other individuals associated with veteran service agencies—with brief training and limited implementation support. Delivery considerations also required the identification of referral pathways for participants who wanted further mental health treatment opportunities. This would feasibly enable the integration of the program into different community-facing organizations and linkages with mental health services outside of the organizations.

### Design and Development of the CPSS Prototype

Based on these considerations, we developed an initial CPSS program prototype from August to September 2019 consisting of a single session with PSS content that could be delivered to a small group of 5–10 participants. The intervention could be facilitated by a single provider or a provider pair. Intervention components included: (1) introduction and psychoeducation; (2) mental health symptom self-assessment; (3) safety identification; (4) active skill building in cognitive coping; and (5) finishing steps. We describe each of these components.

**1. Introduction and psychoeducation.** Consistent with most group interventions, providers begin with introductions, provide personal testimonials about their own experience benefiting from MHPSS interventions, and lead the group in agenda-setting and group expectations. The psychoeducation component includes an introduction to stress—including group identification and normalization of situations in daily life that can be stressful and identification/normalization of common psychosocial impacts of stress. The psychoeducation component also includes brief information from brain science, encouraging the belief that every person has the ability to change the way their brain works and develop the skill they will learn through the intervention. The explicit emphasis on brain science in CPSS meets the interests of Ukrainian veterans in the scientific basis for the intervention while also fostering a growth mindset.^19^

**2. Mental health symptom self-assessment.** The CPSS program includes a self-administered tool that participants complete to assess their stress-related symptoms and to aid in the referral to mental health treatment when needed. Participants are also encouraged to revisit the assessment after the program to determine their potential support needs. The tool was developed from a locally validated mental health assessment^20^ used during CETA.

The CPSS program includes a self-administered tool that participants complete to assess their stress-related symptoms and to aid in the referral to mental health treatment when needed.

**3. Safety identification.** In addition to identifying common psychosocial symptoms of stress, the self-assessment screens for current thoughts of harm to self or others. The CPSS provider normalizes that when people are feeling stressed, they may also have thoughts about harm. The provider emphasizes the need to ensure everyone’s safety and carries out safety planning for those identified as high risk.^21^ From the outset, we also included emergency services as part of our safety protocols, as well as referral to the 24-hour veterans’ helpline “Lifeline.”

**4. Active skill-building in cognitive coping.** We included the CETA element of cognitive coping based on results from the prior stakeholder analysis and CETA trial implementation. In the CETA skill of Thinking a Different Way–Part 1, participants are taught about thoughts, feelings, and behaviors and how they are connected. Using common everyday life stressors as examples, participants practice identifying and changing their unhelpful thoughts and tracing the impact of these changes on their feelings and behaviors.

**5. Finishing steps.** Before ending the group session, providers summarize key points from the program, link the active skill-building back to psychoeducation and brain science, remind participants of referral options and resources, encourage practice of the new skills, and share that a provider will conduct a follow-up contact with them in a few weeks.

Consistent with the CETA approach, when the CPSS intervention design was complete, we developed an intervention manual divided into discrete steps with plain language scripts to support training lay providers to conduct the intervention with fidelity. Providers were trained using an apprenticeship-based approach.^17^ At the initial training, trainee providers first read the scripts together with real-time discussion suggestions for improvement followed by multiple rounds of role-play and paired practice.

### Round 1 Implementation: Initial Testing and Iterative Refinement in a Neutral Setting

The first round of CPSS training, implementation, and iterative refinement took place September 26–29, 2019. The focus of this initial round was to observe, gather feedback, and refine the training materials, intervention materials, and delivery processes.

#### Setting and Participants

Nine experienced local CETA providers—including 1 veteran and 2 spouses of veterans—were identified for CPSS training. Engaging experienced providers ensured they were already familiar with the relevant CETA clinical content and able to provide feedback on delivery processes in the group format; these individuals were also identified as potential future trainers to support program sustainability. Round 1 CPSS group participants were a convenience sample of adult veterans and family members of veterans who were recruited through social media, MOVA contacts, and local veteran service organizations that were actively supporting our intervention development efforts. Staff from these veteran service organizations were also invited to participate, with the rationale that these providers were familiar with existing services and veterans’ needs and could potentially be involved in delivering CPSS. CPSS sessions were held in university classrooms in downtown Kyiv (i.e., a neutral location but not somewhere veterans and family members would naturally be attending services) and were observed by members of the U.S.-based CETA clinical team with the support of local interpreters. All participants were informed that the CPSS session was being offered as part of a development process and that they would be invited to a feedback discussion immediately after the session. The feedback discussions were designed as a standard program development activity; as such, while participants were informed of their purpose and scope, they were not asked to provide formal informed consent.

#### Procedures

The first day of provider training covered all CPSS content, with trainees actively engaged in providing feedback and suggestions for improvements. The second day of training involved practice groups, followed by the facilitation of 4 CPSS sessions by provider pairs in the evening. After completing the sessions, the CPSS providers and clinical team left the room, and independent focus group facilitators led feedback discussions with each of the CPSS group participants. Questions covered which aspects of the materials participants thought veterans and their families would like and find useful, as well as which aspects were confusing, too complicated, or made participants feel uncomfortable. Participants were also asked whether they thought veterans and their family members would be likely to recommend the session to others and why.

The next day, the CETA clinical team reviewed the participant feedback, held a debriefing discussion with the providers, and then together incorporated changes into the session materials. The CPSS providers were then retrained on these updates before leading 4 additional CPSS sessions. Again, the CETA clinical team observed the sessions, and participants provided feedback after the same process previously described. Feedback was reviewed, providers debriefed, changes made, and providers were retrained the next day. Across the 2 days of CPSS session implementation, groups of different sizes and composition (whether participants were only veterans or veterans and family members together) were tested. Variations in whether participants did the practice activities alone or within small breakout groups were also evaluated, as were different examples of stressful situations.

### Round 2 Implementation: Field-Testing in a Community Setting

After completion of the iterative intervention development, a package of accompanying implementation supports was developed. This included a complete CPSS training guide, as well as facilitator and supervisor fidelity checklists. These implementation tools were intended to support local trainers-in-training in the supervision and training of community-based providers in CPSS delivery. The revised CPSS program was implemented with 3 additional groups on October 18–19, 2019. The focus of this second round of implementation was to field-test the revised, quasi-finalized CPSS program and related implementation processes in more real-world conditions.

#### Setting and Participants

Six new CPSS providers were trained to lead the sessions in round 2 implementation. All were current providers of other veteran-related services and were referred for CPSS training by their organizational leads as part of ongoing partnership and service integration efforts. CPSS group participants were adult veterans and family members of veterans recruited using the same approach described in round 1. CPSS sessions were held with the veterans and family members at community-based veterans’ service organizations in Kyiv (i.e., sites where future intervention integration was planned).

#### Procedures

Each of the 3 CPSS trainee pairs completed a 2-day training that incorporated updated materials as well as additional practice in group facilitation. Trainee pairs were partnered with a newly certified CPSS trainer (i.e., CETA providers who received CPSS training in round 1, followed by subsequent training of trainers) to cofacilitate a group. CPSS session participants were again invited to stay after the session to provide feedback to an independent focus group facilitator, and a debrief with the providers was held using the same discussion format described in round 1. The feedback was intended to provide any further final input into the CPSS program before formal piloting. Because some program elements (e.g., a newly introduced 1-week follow-up call and referrals to CETA) were extended after the immediate feedback session and to ascertain longer-term perceptions of the program, we attempted to recontact participants by phone 3 months after the sessions in this round to follow up on their experiences. However, we decided this in later discussions and had not advised participants to expect this.

### Formal Pilot Evaluation

The formal pilot study took place in March 2020. This pilot was not powered to measure impact but rather focused on collecting initial data in the context of field-testing implementation and study procedures for a subsequent efficacy trial.

#### Setting and Participants

Paired facilitators led 3 in-person CPSS sessions (1 group of 4 and 2 groups of 10). CPSS sessions were held in veterans’ rehabilitation centers and community centers that ran programs for veterans in the Kyiv region. Participants were a new convenience sample of veterans and adult family members recruited through social media and veterans’ service organizations. Participants were invited to attend the session regardless of their interest in study participation. At the end of the session, participants who were willing to participate in further study-related contacts gave their informed consent; 19 (79%) gave their consent to participate in the pilot research.

#### Data Collection and Procedures

During the CPSS session, participants completed the 7-domain self-assessment (Box). Responses were provided on a Likert-style scale considering frequency of experience over the past 2 weeks. Self-assessment scores were totaled for each domain. Higher scores reflected greater difficulties, with the exception of positive communication, for which the direction was reversed.

BOX Domains of the Self-Assessment Completed by CETA Psychosocial Support Participants

1. Mental health symptoms (21 items)^a^
2. Functional impairment (8 items)^a^
3. Safety risk (2 items)^a^
4. Alcohol use (3 items)^b^
5. Social disconnect (5 items)^c^
6. Aggression (4 items)^d^
7. Positive communication (2 items)^d^

^a^ From the previously validated mental health assessment tool used in the Common Elements Treatment Approach in Ukraine.^20^

^b^ Source: AUDIT-C.^22^

^c^ Source: Social Connectedness Scale.^23^

^d^ Source: Conflict Tactics Scale.^24^

After the CPSS session, anyone who indicated current safety risk was contacted and provided with an immediate CETA referral. All others were contacted again within 1 week to discuss their self-assessment results and provide guidance for practicing the coping skills covered in the CPSS session. One month later, participants were recontacted to complete another self-assessment. Those reporting mental health symptoms above a standard cutoff (≥13/63) at the 1-month follow-up were referred to CETA.

At the 1-month follow-up, participants also completed the Mental Health Implementation Science Tools Consumer version,^25^^,^^26^ an implementation research measure that assesses consumer perceptions of program adoptability (4 items), acceptability (9 items), appropriateness (6 items), feasibility (8 items), and accessibility (8 items). Participants responded to statements related to each implementation domain, such as whether they would use CPSS in the future if needed, using Likert-style response options from 0 to 3 (“no,” “somewhat no,” “somewhat yes,” and “yes”). Average scores were generated for each implementation domain, and individual item scores were also explored. The Mental Health Implementation Science Tools have shown good psychometric properties in multiple countries, with demonstrated validity of the client instrument in Ukraine.^25^^,^^26^

The rationale for a 1-month follow-up period for both regular programmatic practice and study-related data collection was that this provided sufficient time to practice and apply the mental health promotion skills covered in the CPSS session and experience any related psychosocial benefits while minimizing any delay in referral for individuals needing additional support. It is important to note that this 1-month follow-up assessment and referral occurred during the initial months of the COVID-19 pandemic.

### Ethical Approval

The pilot received approval of human subjects research from the Johns Hopkins Bloomberg School of Public Health Institutional Review Board (JHSPH IRB #9782).

## RESULTS

### Round 1 Implementation: Initial Testing and Iterative Refinement in a Neutral Setting

A total of 62 participants (33 women, 26 men, 3 not reported; 38 veterans, 9 family members of veterans, 15 providers) attended the 8 initial CPSS sessions, which averaged 109 minutes in duration. Based on self-assessment responses, 44 (71%) required post-session follow-up for further safety assessments and/or to provide referrals to the full CETA intervention. Changes made to the materials during the iterative development stage included content, process, and group facilitation adjustments.

#### Content Feedback

Our feedback sessions included adjustments and additions of “everyday stressor” scenarios that better reflected the experiences of veterans and family members. An example of an updated scenario was being refused a free bus ride, to which they are entitled. For the cognitive coping skill-building component, several participants struggled to differentiate between thoughts, feelings, and behaviors, leading to addition of a step that briefly provided an overview of these 3 concepts. Some participants raised concerns that the session topic, “thinking a different way,” could be misunderstood as simply trying to tell people to “think positively” without recognizing their trauma and lived experiences, which led to adjustments in how the skill-building component was introduced. The providers were very positive about adding the brain science component and felt it was a perfect explanation as to why we teach “thinking in a different way” from CETA and why it is important to practice. Finally, they suggested adjusting the practice activity to include time for personal reflection.

Our feedback sessions included adjustments and additions of “everyday stressor” scenarios that better reflected the experiences of veterans and family members.

#### Process Feedback

A key lesson from the participant feedback was the need to manage expectations. Many participants expressed feeling as though there was a lack of clarity around the purpose of the session and whether this was intended to be the first in a series. Some felt that the intervention was too simple and nothing new; others felt that they didn’t fully understand the skill and would need additional sessions for practice. Some participants wanted the opportunity to go further into processing traumatic experiences. Based on this feedback, the introduction steps were revised to provide additional clarity around the goals and agenda for the session—emphasizing the need to practice on their own to build competency in changing thoughts—and how the session was intended to fit within a larger system that could include referral for mental health treatment. We also added a 1-week follow-up call for all participants (not just those requiring immediate referrals) to check in on their use of the new skill over the past week and to provide correction if necessary.

Adjustments were also made to the length and balance of the session. Both providers and participants felt the sessions were too long and very information-heavy, with not enough time for skills practice. Observers noted that in some cases, providers spent a lot of time adding extra information about brain science. In response to the process feedback, initial steps were shortened and streamlined, providers did more role-plays with trainers to manage “add-ins,” and the intervention was designed to include a natural stopping point for a midway break.

#### Group Facilitation Feedback

Veterans appreciated having a veteran CPSS provider, noting that this was not a necessary requirement but that it promoted trust. Providers reported that facilitating more homogenous groups (e.g., all veterans or all providers) was easier. There had been some uncertainty as to whether participants should complete the second practice activity alone or in groups, and it was determined that group facilitation was preferable. Providers also felt it would be helpful to have more tools and handouts, which were developed after this initial round of implementation was completed. More generally, observers noted that further training on group facilitation skills was also necessary. This led to substantial additional training in group facilitation built into the training program for all future CPSS providers.

Veterans appreciated having a veteran CPSS provider, noting that it promoted trust.

### Round 2 Implementation: Field-Testing in a Community Setting

The revised program was field-tested with 23 people (11 male, 12 female; 9 veterans, 14 family members) over 3 sessions averaging 111 minutes in duration (inclusive of the newly added midsession break). Feedback from both providers and participants indicated positive reception of the revised materials and processes, with no notable suggestions for further revision of the intervention content. With the additional training focus on group facilitation, the newly trained providers were able to satisfactorily manage group dynamics. Based on our experience, we continued to refine tracking and fidelity monitoring tools after the field test with the new providers. Seven participants were successfully recontacted 3 months later; 5 reported that they had used the cognitive skill covered in the CPSS session and found it useful.

### Pilot Evaluation

Twenty-four participants attended 1 of 3 CPSS pilot sessions, of which 8 (33%) were veterans and 16 (67%) were family members; 14 (58%) were women, and the median participant age was 37 years (interquartile range: 33–45 years). Of the pilot participants, 8 women and 2 men completed follow-up 1 month after their CPSS session. Duration of pilot sessions averaged 133 minutes, with the additional time largely due to delayed starts and the addition of study procedures.

**FIGURE 1:**
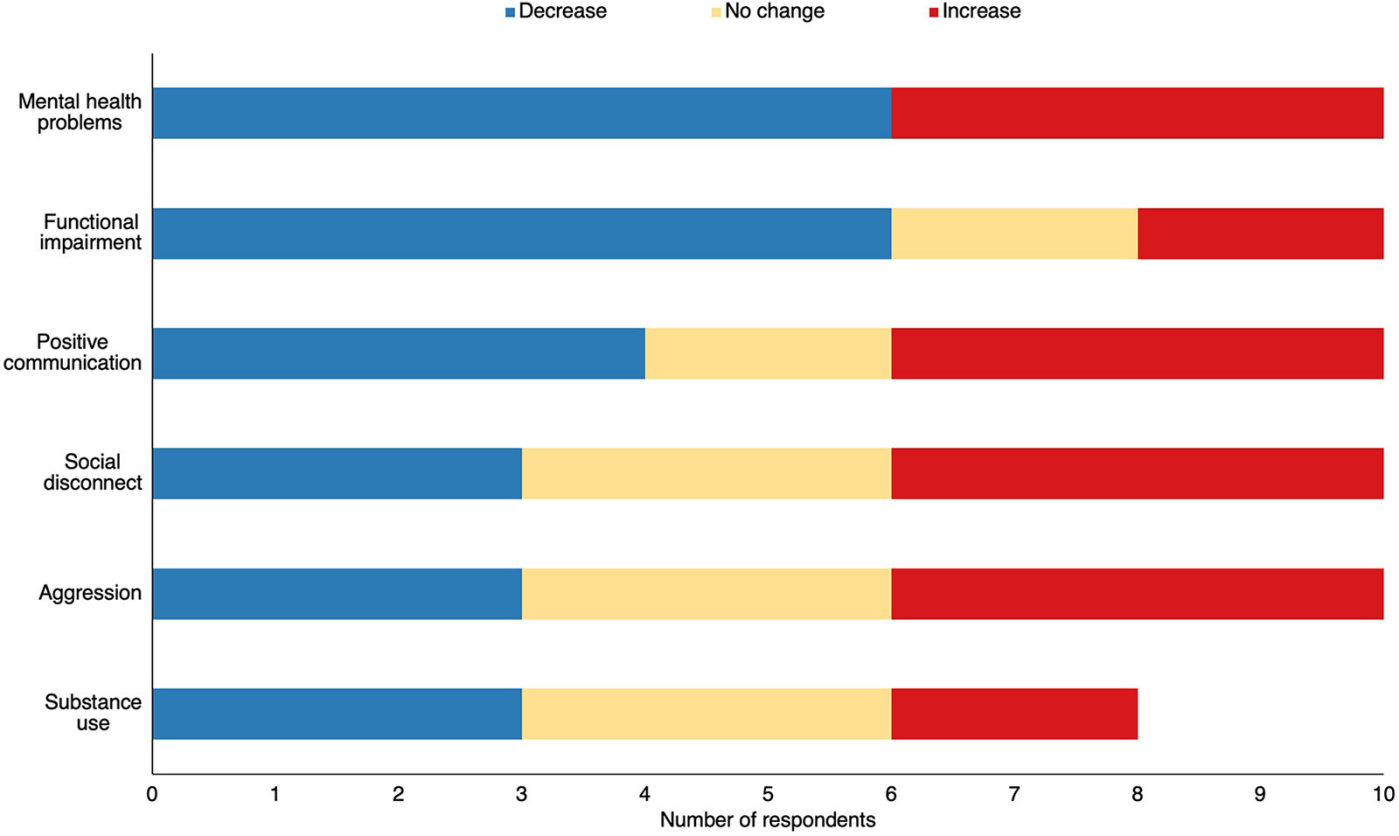
Change in Outcomes at 1 Month for Common Elements Treatment Approach Psychosocial Support Program Participants in Ukraine

The small sample size precludes statistical tests of differences in outcomes from baseline to 1 month; however, trends suggest improvements (i.e., decrease in scores) in mental health symptoms (“problems”) and functional impairment (Figure 1). Two participants who continued to experience substantial symptoms at follow-up were referred to the full CETA counseling program for further evaluation and treatment.

**FIGURE 2:**
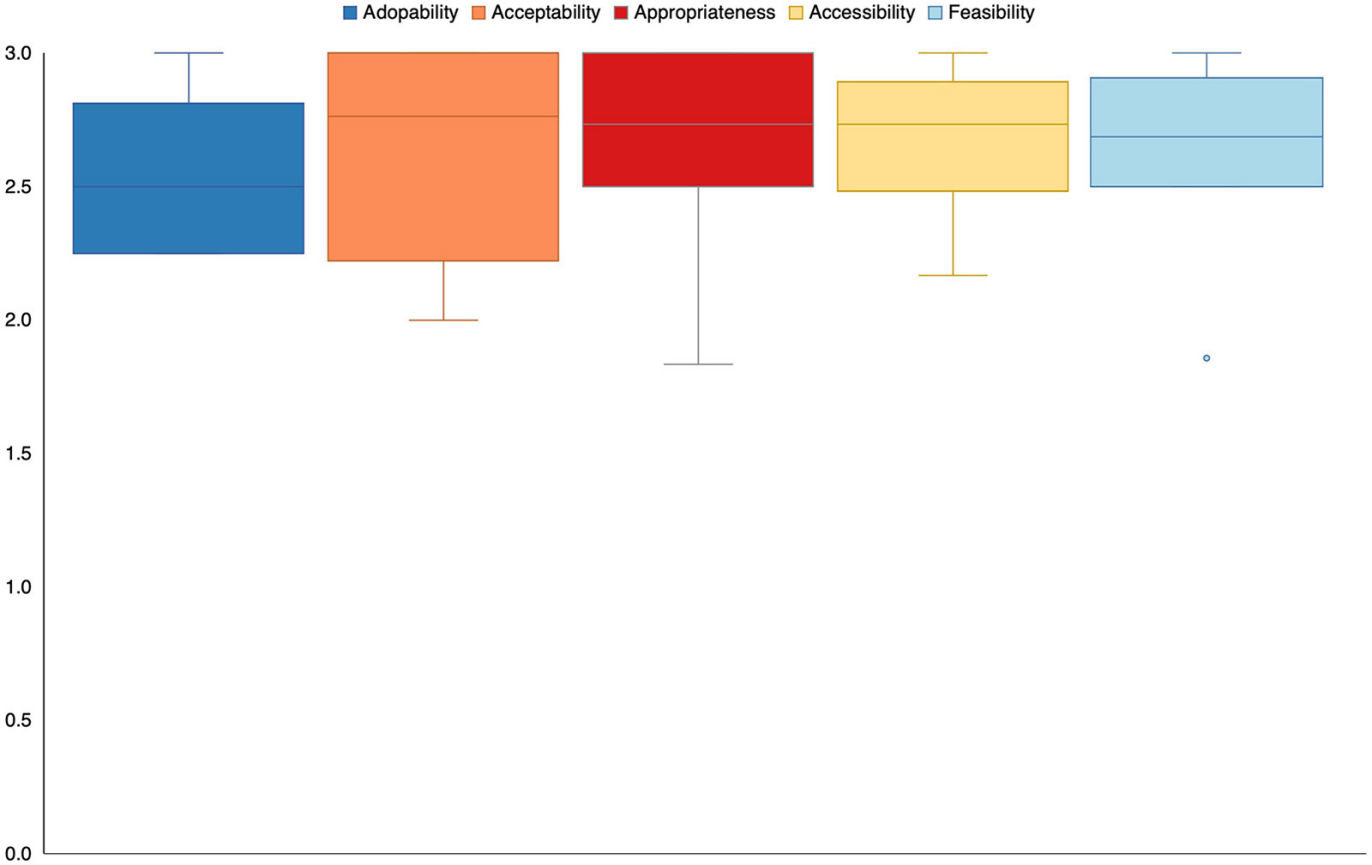
Implementation Outcome Distributions for Common Elements Treatment Approach Psychosocial Support Program in Ukraine^a^ ^a^ N=10; Responses provided on 0–3 scale with high scores indicating a more positive domain rating. Plots display score distributions; boxes indicate interquartile range (i.e., center 50% of scores), and dot indicates outlying value.

In exploring the implementation domains (Figure 2), participants rated the CPSS highly positively, with nearly all participants responding “somewhat yes” or “yes” to questions related to each implementation domain. When exploring variations on individual implementation questions, 2 participants responded “no” or “somewhat no” to the following statements: whether participants were easily able to get away from duties to attend CPSS, whether participants had used skills learned in CPSS, and whether participants felt they had the emotional support to complete CPSS.

## DISCUSSION

Through stakeholder engagement and an iterative development process, we developed an acceptable and potentially effective brief PSS prevention and promotion program that could introduce MHPSS concepts and skills and act as a gateway for referral to more intensive mental health treatments for those who need it. The development process resulted in a program with content and implementation strategies tailored to Ukrainian veterans and their families. One example of incorporating stakeholder feedback was prioritizing training veteran service providers as CPSS providers based on both preferences identified during our formative work and our partnerships with service organizations.

Because the content of the CPSS program was derived from the CETA intervention, which emphasizes the use of common evidence-based intervention elements, the CPSS includes many elements, such as psychoeducation, engagement, active skill-building, and promotion of adaptive thinking, that are consistent with other brief interventions.^19^^,^^27^^,^^28^ The CPSS program expands the component of psychoeducation, which in most programs focuses on introducing mental health disorders and normalizing symptoms. In the CPSS program, we empower participants to understand their own mental health through the use of a self-assessment, which also serves to identify individuals at high risk for suicide and those who should be referred for further mental health intervention services. Use of screeners for data-based decision-making is an important element of many brief intervention and referral strategies and is consistent with a public health approach to improve outcomes across a range of service systems.^29^^–^^31^ Given our previous experience with CETA in Ukraine, we had anticipated a high degree of skepticism regarding screening and were surprised at the readiness with which participants approached the screener. Although this could reflect a convenience sample, we also felt it indicated a potential shift in veterans’ comfort in discussing mental health in recent years.

The CPSS program empowers participants to understand their own mental health through the use of a self-assessment, which also screens for suicide risk and further referral needs.

The components of the CPSS program (psychoeducation, assessment review, safety, “thinking in a different way”, and finishing steps) needed relatively little modification. However, over the course of the development process, we made several changes that addressed the need for greater contextualization of the intervention, more attention to group dynamics and facilitation skills, and integration of procedures for follow-up conversations. As these changes arose even during initial implementation by skilled CETA providers, they highlighted the critical need to attend to what may be considered common or nonspecific therapeutic factors of programming, such as provider competence, therapeutic alliance, and attention to procedures and contextual factors.^32^ There is a need for careful attention to training and support for these programmatic factors, especially when working with lay providers.^32^

CPSS was developed to be part of a broader-tiered, community-based mental health program as a brief, first-tier universal prevention and promotion session that can be implemented in a range of nonclinical settings. In addition to supporting mental health promotion, the CPSS program is intended to serve as an entrance point for referral and connection to a full psychotherapy program such as CETA or psychiatric services for participants who could benefit from more intensive mental health interventions because of high-risk symptoms such as suicidality and/or a high burden of mental health symptoms. With 44 participants (across all implementation stages) requiring post-session follow-up for further safety assessments and/or to provide referrals to the full CETA intervention, it is clear that lower-tiered psychosocial supports need to be part of a multitiered service system rather than stand-alone programs.

This article is an important contribution to a description of the iterative process of the CPSS program development, following consensus-based guidelines for reporting on intervention development.^33^ The rationale, development and revision processes, and findings along the way are rarely presented in pilot and efficacy trials, despite the importance of unpacking the “black box” of intervention development.^34^ Beyond the value gained by describing this project, we hope that sharing the detailed description of our development process will assist others in developing a replicable mental health treatment approach.

While improved reporting of intervention development is critical to building the evidence base, an initial description alone is insufficient because of how rapidly changing contexts often lead to ongoing adaptation during delivery. As such, it is also critical to examine the extent to which interventions are capable of adaptation while maintaining fidelity to core components. After completion of this development process, in early 2020, we initiated a randomized controlled trial to evaluate the effectiveness of the CPSS program in reducing the burden of moderate-to-low levels of mental health symptoms and increasing uptake of the CETA psychotherapy intervention for those with moderate to high symptoms. Due to the COVID-19 pandemic, we adapted CPSS for virtual delivery during the trial. The trial was completed just before the Russian invasion of Ukraine in February 2022 and will be the subject of a second, forthcoming article. Additionally, since the start of the Russian invasion, we have supported previously trained CETA providers to continue to provide CPSS and full CETA services through telehealth and virtual delivery platforms. Their capability to provide MHPSS content across the continuum has been an asset given the high need for more substantial mental health supports for Ukrainians who remained in the country. Anecdotally, we have found the CPSS program to be highly adaptable within this rapidly changing intervention environment, with our providers leading groups in a range of challenging circumstances.

### Limitations

A couple of limitations need to be noted, including the relatively small number of veterans who participated in the formal piloting compared with veteran family members. In the earlier phases of implementation, more than 50% of the participants were veterans, which provided rich and useful information throughout the development process. But given the small number who participated in the final pilot, further information on the effectiveness and acceptability among veterans is needed. Follow-up during our formal pilot testing period was also highly impacted by the onset of the COVID-19 pandemic, which led to a less robust follow-up sample than anticipated. It is possible that the small number of participants who completed their follow-ups reflected a biased sample, though we note that participants in that group included those who had shown improvement and participants who required referral for additional care. In terms of modifying the program for virtual delivery, adaptation was done as the program was being rolled out for the larger trial. No additional content adaptations were needed, but training providers to deliver the program virtually and supporting participants in this new online world required patience and practice.

## CONCLUSIONS

Despite the growth of evidence and availability of mental health programs in many low- and middle-income countries, there remains a lack of psychosocial programming that is specifically designed to increase knowledge and uptake of mental health services among those who might benefit from them. Most psychosocial programs focus on prevention and promotion and exist as stand-alone programs, while many people, particularly in conflict and humanitarian crisis settings, often need more than support and education. The CPSS program was developed with iterative feedback from Ukrainian veterans and their families to normalize stress and distress, develop some initial skills in cognitive coping, and provide referral to more intensive mental health services for those at high risk for suicide and/or with a higher burden of symptoms. Brief psychosocial programs can be a valuable component of a larger, multitiered MHPSS continuum of care that supports further referral as needed.

## Notes

**Peer Reviewed**

**First published online:** May 18, 2023.
